# Correction

**DOI:** 10.1111/cas.13258

**Published:** 2017-04-26

The authors would like to draw the reader's attention to errors in the following article:

Tomoki Y, Hamanaka M, Babaya A, Kimura K, Kobayashi M, Fukumoto M, Tsukamoto K, Noda M, Matsubara N, Tomita N, Sugihara K. Management strategies in Lynch syndrome and familial adenomatous polyposis: a national healthcare survey in Japan. *Cancer Sci* 2017; **108:** 243–9.

The correct funding information is shown below:

Japanese Society for Cancer of the Colon and Rectum

Additionally, all occurrences of ‘Japan Society of Colorectal Cancer Research’ in the article should have changed to ‘Japanese Society for Cancer of the Colon and Rectum’.

The authors apologize for these errors and any confusion these may have caused.

